# Supplementary material for: Comparative Genomics of Pseudomonas aeruginosa Strains Isolated from Different Ecological Niches
Source: Antibiotics (Basel). 2023 May 7;12(5):866. doi: 10.3390/antibiotics12050866 (PMC10215170; doi:10.3390/antibiotics12050866)
Supplement: Supplementary file 1 [file antibiotics-12-00866-s001.zip › antibiotics-2301852-supplementary.pdf]

Table S1. General features of the drafts of four *P. aeruginosa* strains isolated from the Mexican Hospital.

Features of the genomes	PE21	PE52	PE63	PE83
Total contigs	101	47	26	103
G+C content (bp)	65.47	66.14	66.57	65.51
Genome size (bp)	7,388,095	6,786,596	6,235,658	7,342,540
Number of genes	6,961	6,348	5,735	6,924
Number of coding sequences (CDS)	6,884	6,274	5,662	6,847

The number of genes and CDS was determined based on the Prokka annotations results.

Table S2. General features of *P. aeruginosa* genomes included in this study.

STRAIN	ACCESSION NUMBER	ST	ISOLATION SOURCE	COUNTRY	GENOME SIZE (bp)	RESISTANCE GENOTYPE
UCBPP-PA14	CP000438.1	253	-	-	6,537,648	*crpP, fosA, catB7, bla*$_{OXA-488}$, *bla*$_{PAO}$, *aph(3')-IIb*
PAO1	NC_002516.2	549	Burn wound isolate	-	6,264,404	*aph(3')-Iib, catB7, bla*$_{OXA-50}$, *bla*$_{PAO}$, *fosA*
PE21	JARDUV000000000	167	Sputum	Mexico	7,398,559	*aph(3'')-Ib, aac(6')-II, aadA1b, aph(3')-Via, aph(3')-Iib, aadA6*, *aac(6')-33*, *sul1*, catB7, catA1, *bla*$_{GES-32}$, *bla*$_{OXA-2}$, *bla*$_{IMP-62}$, *bla*$_{OXA-494}$, *bla*$_{PAO}$, *fosA*
PE52	JARDUU000000000	2731	Urine	Mexico	6,775,738	*aph(3')-Iib*, aadA1b, *fosA*, *sul1*, catB7, *bla*$_{PAO}$, *bla*$_{IMP-56}$, *bla*$_{OXA-2}$, *bla*$_{OXA-396}$
PE63	JARDUW000000000	549	Urine	Mexico	6,219,535	*aph(3')-Iib, catB7*, *bla*$_{OXA-50}$, *bla*$_{PAO}$, *fosA*
PE83	JARDUX000000000	167	Urine	Mexico	7,358,407	*qnrVC1, aac(6')-II, aph(3')-Via, aadA1b, aadA6, aph(3'')-Ib, fosA*, *sul1*, *dfrA15*, *catB7*, *catA1*, *bla*$_{PAO}$, *bla*$_{OXA-2}$, *bla*$_{OXA-494}$, *bla*$_{IMP-62}$
97	NZ_CP031449.2	234	Urine	Ghana: Kumasi	6,925,889	*crpP, qnrVC1, aadA1, aac(6')-Ib3, aac(6')-Ia, aph(3')-II, aadA10, fosA, dfrB5, sul1, catB7, arr-2, bla*$_{IMP-1}$, *bla*$_{OXA-486}$, *bla*$_{DIM-1}$, *bla*$_{OXA-10}$, *bla*$_{PAO}$, *bla*$_{OXA-129}$
A-I-1	NZ_CP060243.1	111	Urine	Francia: Rouen	7,087,087	*crpP, aph(3')-Iib, aac(6')-Ib3, aadA2b, fosA, sul1, catB7, bla*$_{PAO}$, *cmlA2*, *bla*$_{OXA-9}$, *bla*$_{OXA-395}$, *bla*$_{CARB-2}$, *aacA(6')-Ib*
B-I-1	NZ_CP060242.1	235	Urine	France: Rouen	6,935,851	*aadA11, aph(3')-Iib, aac(6')-Ib3, ant(2'')-Ia, fosA, sul1, dfrB1, bla*$_{OXA-488}$, *bla*$_{PAO}$, *bla*$_{OXA-35}$
CCUG 51971	NZ_CP043328.1	235	Urine	Sweden: Solna	7,012,798	*crpP, fosA, aph(3')-Iib, aac(6')-Ib3, sul1, catB7, arr-7, bla*$_{PAO}$, *bla*$_{OXA-35}$, bla$_{VIM-4}$, bla$_{OXA-488}$
C-I-1	NZ_CP060241.1	3227	Urine	France: Rouen	6,325,392	*aph(3')-II, catB7, bla*$_{PAO}$, *bla*$_{OXA-50}$, *fosA*
F5677	NZ_CP026680.1	111	Urine	USA: New York	6,645,227	*crpP, aph(3'')-Ib, aph(3')-II, aph(6)-Id, catB7, bla*$_{OXA-395}$, *bla*$_{PAO}$, *fosA*
G-I-1	NZ_CP060240.1	232	Urine	France: Rouen	6,480,192	*crpP, aph(3')-IIb, catB7, bla*$_{PAO}$, *bla*$_{OXA-396}$, *fosA*
IMP-13	C: NZ_CP034354.1<br>P: NZ_CP034355.1	621	Urine	Belgium	C: 7,047,704<br>P: 130,306	*crpP, fosA, aph(3')-Iib, aac(6')-Ib4, aph(3')-VI, sul1, catB7, bla*$_{OXA-914}$, *bla*$_{IMP-13}$, *bla*$_{PAO}$

NCGM257	NZ_AP014651.1	357	Urine	Japan	7,090,694	crpP, aadA2, ant(2'')-Ia, aph(3')-IIb, fosA, sul1, tet(G), catB7, bla$_{\text{IMP-1}}$, bla$_{\text{OXA-4}}$, bla$_{\text{OXA-846}}$, bla$_{\text{PAO}}$, ~~floR2~~
PA1088	NZ_CP015001.1	277	Urine	Brazil: Sao Paulo, SP	6,721,480	crpP, fosA, aadA7, aph(3')-Iib, aac(6')-Ib, rmtD1, sul1, cmx, catB7, bla$_{\text{OXA-56}}$, bla$_{\text{PAO}}$, bla$_{\text{OXA-494}}$, bla$_{\text{SPM-1}}$
PB353	C: NZ_CP025051.1<br>P: NZ_CP025052.1	Unknown	Urine	USA: New York	C: 6,437,515<br>P: 59,923	aph(3')-Iib, catB7, bla$_{\text{PAO}}$, bla$_{\text{OXA-50}}$, fosA, aac(6')-Ib, aac(6')-Ib4, sul1, bla$_{\text{TEM-1B}}$, bla$_{\text{OXA-101}}$, bla$_{\text{CTX-M-30}}$
PSE6684	NZ_CP053917.1	773	Urine	South Korea	6,924,367	qnrVC1, aac(6')-Ib, fosA, tet(G), aadA, sul1, aph(3')-Iib, rmtB4, catB7, arr-2, bla$_{\text{NDM-1}}$, bla$_{\text{PAO}}$, bla$_{\text{OXA-796}}$, bla$_{\text{OXA-395}}$
ST773	NZ_CP041945.1	773	Urine	USA: Houston	6,835,731	qnrVC1, rmtB4, aph(3')-Iib, aadA, fosA, sul1, tet(G), catB7, bla$_{\text{NDM-1}}$, bla$_{\text{OXA-395}}$, bla$_{\text{PAO}}$
PA790	CP075176.1	773	Urine	India: Lucknow	6,932,250	qnrVC1, rmtB, aph(3')-Iib, fosA, sul1, tet(G), catB7, aadA, bla$_{\text{OXA-395}}$, bla$_{\text{NDM-1}}$, bla$_{\text{PAO}}$
ZBX-P12	CP061779.1	233	Urine	Lebanon:Beirut	7,080,089	crpP, aac(6')-Ib, fosA, aac(6')-Iic, aph(3')-Iib, aadA2, sul1, cmlA1, catB7, bla$_{\text{OXA-4}}$, bla$_{\text{IMP-15}}$, bla$_{\text{OXA-486}}$, bla$_{\text{PAO}}$
PAM68	CP065948.1	1639	Urine	China: Shaanxi	6,560,248	crpP, aph(3')-Iib, catB7, bla$_{\text{OXA-486}}$, bla$_{\text{PAO}}$, fosA
PAS6	CP065947.1	1639	Urine	China: Shaanxi	6,742,928	crpP, aph(3')-Iib, catB7, bla$_{\text{OXA-50}}$, bla$_{\text{PAO}}$, fosA
PAG7	CP065374.1	1639	Urine	China: Shaanxi	6,715,242	crpP, fosA, catB7, bla$_{\text{OXA-50}}$, bla$_{\text{PAO}}$, aph(3')-Iib
SE5429	CP054845.1	235	Urine	China: Jiangsu	7,103,853	crpP, fosA, aadA1, aac(3)-Iid, aph(3')-VI, aph(3')-Iib, sul1, cml, catB7, bla$_{\text{PAO}}$, bla$_{\text{VEB-3}}$, bla$_{\text{OXA-488}}$, bla$_{\text{OXA-10}}$
1811-13R031	NZ_CP046061.1	395	Sputum (lung infection)	China	7,344,079	crpP, aph(3')-Iib, catB7, bla$_{\text{OXA-905}}$, bla$_{\text{PAO}}$, fosA
1811-18R001	NZ_CP046060.1	395	Sputum (lung infection)	China	7,343,000	crpP, fosA, catB7, bla$_{\text{PAO}}$, bla$_{\text{OXA-905}}$, aph(3')-Iib
60503	C: NZ_CP041774.1<br>P: NZ_MN208063.1	773	Sputum (pneumonia)	China: Beijing	C: 6,809,062<br>P: 407,628	crpP, aph(3')-Iib, bla$_{\text{OXA-395}}$, bla$_{\text{PAO}}$, fosA, aph(6)-id, aadA1, qnrVC6, sul1, dfrA1, dfrB2c, bla$_{\text{OXA-4}}$, bla$_{\text{DIM-1}}$, catB3, aph(3'')-Ib, aac(6')-Ib
1903031130	CP060392.1	235	Sputum (pneumonia)	China: Henan	6,905,506	fosA, aph(3')-Iib, aph(3')-XV, sul1, aadA6, tet(G), catB7, bla$_{\text{GES-15}}$, bla$_{\text{PAO}}$, bla$_{\text{OXA-488}}$, aac(6')-Ib4
AG1	NZ_CP045739.1	111	Sputum-lungs (Pneumonia)	Costa Rica	7,190,208	crpP, fosA, aph(3')-Iib, aac(6')-29a, aac(6')-29b, sul1, catB7, bla$_{\text{OXA-2}}$, bla$_{\text{IMP-18}}$, bla$_{\text{PAO}}$, bla$_{\text{VIM-2}}$, bla$_{\text{OXA-395}}$
LW	NZ_CP022478.1	1182	Sputum (infection)	China: Beijing	6,824,837	crpP, aph(3')-Iib, catB7, bla$_{\text{OXA-851}}$, bla$_{\text{PAO}}$, fosA
PA19-3047	CP068239.1	235	Sputum (bronchiectasis)	China	6,857,397	crpP, ant(2'')-Ia, aac(6')-Ib, aadA6, aph(3')-Iib, fosA, sul1, dfrA5, cmx, catB3, catB7, bla$_{\text{PER-4}}$, bla$_{\text{PAO}}$, bla$_{\text{OXA-488}}$, bla$_{\text{OXA-17}}$, bla$_{\text{OXA-129}}$, aac(6')-Ib'
PASGNDM345	NZ_CP020703.1	308	Sputum	Singapore	6,893,164	aadA, aph(3'')-Ib, aph(3')-Iib, aac(6')-Il, aph(6)-Id, aac(3)-Id, fosA, msr(E), sul2, dfrB5, floR, catB7, bla$_{\text{OXA-488}}$, bla$_{\text{PAO}}$, bla$_{\text{NDM-1}}$, qnrVC1, crpP
PASGNDM699	NZ_CP020704.1	308	Sputum	Singapore	6,985,102	qnrVC1, crpP, fosA, aadA, aac(3)-Id, aph(3')-Iib, aph(3'')-Ib, aph(6)-Id, aac(6')-Il, msr(E), dfrB5, sul2, floR, catB7, bla$_{\text{PAO}}$, bla$_{\text{NDM-1}}$, bla$_{\text{OXA-488}}$
PB350	NZ_CP025055.2	235	Sputum	USA: New York	6,752,906	sul1, dfrA10, fosA, catB7, bla$_{\text{PAO}}$, bla$_{\text{OXA-488}}$, ant(2'')-Ia, aph(3')-Iib
R31	C: NZ_CP061850.1;<br>P: NZ_CP061851.1	Unknown	Sputum	China: chongqing	C: 6,893,878<br>P: 29,402	crpP, aph(3')-Iib, catB7, bla$_{\text{PAO}}$, bla$_{\text{OXA-486}}$, fosA, bla$_{\text{KPC-2}}$
SE5352	CP054843.1	235	Sputum	China: Jiangsu	6,904,218	fosA, aadA6, sul1, aac(6')-Ib4, aph(3')-XV, aph(3')-IIb, tet(G), catB7, bla$_{\text{PAO}}$, bla$_{\text{GES-1}}$, bla$_{\text{OXA-488}}$
SE5357	CP054844.1	244	Sputum	China: Jiangsu	7,043,467	crpP, aph(3'')-Ib, aph(3')-IIb, aac(6')-Ib, aadA5, fosA, sul1, catB7, bla$_{\text{PER-1}}$, bla$_{\text{OXA-101}}$, bla$_{\text{OXA-847}}$, bla$_{\text{PAO}}$, aph(6)-Id

SE5416	C: NZ_CP046404.1<br>P: NZ_MN894887.1	697	Sputum (Infection caused by fracture)	China: Suzhou	C: 6,874,270<br>P: 510,711	*crpP, aph(3')-IIb, aadA1, fosA, sul1, tet(G), catB7, cmlA1*, $bla_{OXA-494}$, bla_{PAO}, *mph(A), qnrS2, tet(G)*, bla_{TEM-1}, bla_{KPC-2}, *qacE, aac(3)-IIId*
SP4371	NZ_CP034369.1	357	Sputum (Bacteraemia)	India	6,937,609	*msr(E), aph(3')-VI , aadA1, ant(2'')-Ia, aac(6')-il, aph(3')-Iib, fosA, sul1, dfrB2, tet(A), catB7*, bla_{OXA-10}, bla_{NDM-1}, bla_{VEB-1}, bla_{PAO}, bla_{PME-1}, $bla_{OXA-846}$, *crpP*
SP4527	NZ_CP034409.1	357	Sputum (Bacteraemia)	India	7,005,215	*msr(E), fosA, aac(6')-II, aadA1, aph(3')-VI, ant(2'')-Ia, aph(3')-Iib, sul1, dfrB2, tet(A), catB7, cmlA1, arr-3*, bla_{PAO}, $bla_{OXA-846}$, bla_{OXA-10}, bla_{NDM-1}, bla_{PME-1}, bla_{VEB-1}, *msr(E), crpP*
SP4528	NZ_CP033439.1	357	Sputum (Respiratory tract infection)	India	6,877,287	*crpP, fosA, sul1, dfrB2, aac(6')-II, ant(2'')-Ia, aadA1, aph(3')-Iib, msr(E), tet(A), catB7*, bla_{NDM-1}, bla_{PAO}, $bla_{OXA-846}$, bla_{OXA-10}, bla_{VEB-1}, bla_{PME-1}
Y31	NZ_CP030910.1	Unknown	Sputum (Pneumonia)	South Korea: Seoul	6,831,076	*crpP, fosA, catB7*, bla_{PAO}, $bla_{OXA-486}$, *aph(6)-Id, aph(3')-Iib, aac(2')-Iia, aph(3'')-Ib*
Y71	NZ_CP030911.1	245	Sputum (Pneumonia)	South Korea: Seoul	6,940,949	*crpP, ant(2'')-Ia, aph(3')-Iib, aph(3')-VI, aac(6')-31, fosA, sul1, catB7, cmx*, bla_{PAO}, $bla_{OXA-494}$, bla_{OXA-1}
Y82	NZ_CP030912.1	111	Sputum (Pneumonia)	South Korea: Seoul	7,106,857	*crpP, ant(2'')-Ia, aph(3')-Iib, aac(6')-31, fosA, sul1, catB7*, bla_{PAO}, $bla_{OXA-395}$, bla_{OXA-1}
ACR22	NZ_CP058331.1	Unknown	Sugarcane	China: Nanning	6,490,021	*fosA, catB7*, bla_{PAO}, $bla_{OXA-486}$, *aph(3')-Iib*
AJ D 2	NZ_CP038661.1	234	Rhizosphere	India	6,281,520	*fosA, catB7*, bla_{PAO}, $bla_{OXA-486}$, *aph(3')-Iib*
B10W	NZ_CP017969.1	308	wastewater	USA: Honolulu	6,723,378	*crpP, aph(3')-Iib, catB7*, bla_{PAO}, $bla_{OXA-488}$, *fosA*
CFSAN084950	NZ_CP045768.1	Unknown	Arugula	USA:GA	6,441,924	*fosA, catB7*, bla_{PAO}, $bla_{OXA-494}$, *aph(3')-Iib*
DN1	C: NZ_CP017099.1;<br>P: NZ_CP018048.1	316	Soil	China	C: 6,641,902<br>P: 317,349	*crpP, aph(3')-Iib, catB7*, bla_{PAO}, $bla_{OXA-395}$, *fosA*
FA-HZ1	NZ_CP017353.1	27	wastewater	China	6,866,790	*crpP, fosA, catB7*, bla_{PAO}, $bla_{OXA-494}$, *aph(3')-Iib*
HS9	NZ_CP030861.1	27	Soil	China: Shanghai	6,876,988	*crpP, fosA, catB7*, $bla_{OXA-494}$, bla_{PAO}, *aph(3')-Iib*
JB2	NZ_CP028917.1	296	Soil	USA: Madison, WI	6,867,314	*crpP, fosA, catB7*, $bla_{OXA-488}$, bla_{PAO}, *aph(3')-Iib*
JT86	NZ_CP062219.1	970	Soil	China: Guangzhou	6,520,277	*fosA, catB7*, bla_{OXA-50}, bla_{PAO}, *aph(3')-Iib*
KRP1	NZ_CP046069.1	27	Methanogenic sludge from a potato processing plant	Belgium: Primeur, Waregem	6,737,396	*crpP, fosA, catB7*, $bla_{OXA-494}$, bla_{PAO}, *aph(3')-IIb*
L10	NZ_CP019338.1	253	halobiotic reed	China: Binzhou	6,661,962	*fosA, catB7*, $bla_{OXA-488}$, bla_{PAO}, *aph(3')-Iib*
M8A1	NZ_CP015647.1	1054	crude oil wastewater	Colombia: Arauca	6,368,297	*fosA, catB7*, $bla_{OXA-396}$, bla_{PAO}, *aph(3')-Iib*
N17-1	NZ_CP014948.1	2362	Soil	China: Haidian, Beijing	6,370,730	*crpP, fosA, catB7*, $bla_{OXA-494}$, bla_{PAO}, *aph(3')-Iib*
Ocean-1155	NZ_CP022526.1	316	Open sea	Pacific Ocean	6,952,237	*crpP, fosA, catB7*, $bla_{OXA-395}$, bla_{PAO}, *aph(3')-Iib*
Ocean-1175	NZ_CP022525.1	316	Open sea	Pacific Ocean	6,943,220	*crpP, fosA, catB7*, $bla_{OXA-395}$, bla_{PAO}, *aph(3')-Iib*
PPF-1	NZ_CP023316.1	Unknown	Dental Unit Water Line	Canada: Montreal	6,930,893	*crpP, fosA, catB7*, $bla_{OXA-908}$, bla_{PAO}, *aph(3')-Iib*
RD1-3	NZ_CP047697.1	257	landfill	China: Yangling	6,397,159	*fosA, catB7*, $bla_{OXA-1026}$, bla_{PAO}, *aph(3')-Iib*
SJTD-1	NZ_CP015877.1	2619	Soil	China: Shanghai	6,243,825	*fosA, catB7*, $bla_{OXA-486}$, bla_{PAO}, *aph(3')-Iib*
TJ2019-017	NZ_CP065866.1	241	Water	China: Tianjin	6,478,778	*crpP, fosA, catB7*, $bla_{OXA-1127}$, bla_{PAO}, *aph(3')-Iib*
YL84	NZ_CP007147.1	169	compost	Malaysia	6,433,441	*crpP, fosA, catB7*, $bla_{OXA-396}$, bla_{PAO}, *aph(3')-Iib*

The letter “C” means Chromosome and “P” means Plasmid

Table S3. Strains with ST167 reported in the MLST database.

STRAIN	ACCESS	ST	COUNTRY	ISOLATION SOURCE	RESISTANCE GENOTYPE
C40	GCF_000480475.1	167	USA	Environmental	*crpP, fosA, catB7, bla*$_{PAO}$*, aph(3')-IIb, bla*$_{OXA-494}$
AZPAE13872	GCF_000795625.1	167	Mexico	Unknown	*fosA, aadA1b, aph(3')-VIa, aac(6')-II, aph(3')-IIb, sul1, catB7, bla*$_{PAO}$*, bla*$_{OXA-494}$*, bla*$_{IMP-15}$
AZPAE14687	GCF_000794515.1	167	Mexico	Respiratory tract infection	*aac(6')-II, fosA, aadA6, aph(3')-IIb, aadA1b, sul1, catA1, catB7, bla*$_{PAO}$*, bla*$_{OXA-494}$*, bla*$_{IMP-62}$*, bla*$_{OXA-2}$
PAE0095	GCA_021693455.1	167	Unknown	-	*crpP, fosA, catB7, bla*$_{PAO}$*, aph(3')-Iib, bla*$_{OXA-494}$
PE21	JARDUV000000000	167	Mexico	Sputum	*aph(3'')-Ib, aac(6')-II, aadA1b, aph(3')-Via, aph(3')-Iib, aadA6*, *aac(6')-33*, *sul1*, catB7, catA1, *bla*$_{GES-32}$, *bla*$_{OXA-2}$, *bla*$_{IMP-62}$, *bla*$_{OXA-494}$, *bla*$_{PAO}$, *fosA*
PE83	JARDUX000000000	167	Mexico	urine	*qnrVC1, aac(6')-II, aph(3')-Via, aadA1b, aadA6, aph(3'')-Ib, fosA*, *sul1*, *dfrA15*, *catB7*, *catA1*, *bla*$_{PAO}$, *bla*$_{OXA-2}$, *bla*$_{OXA-494}$, *bla*$_{IMP-62}$

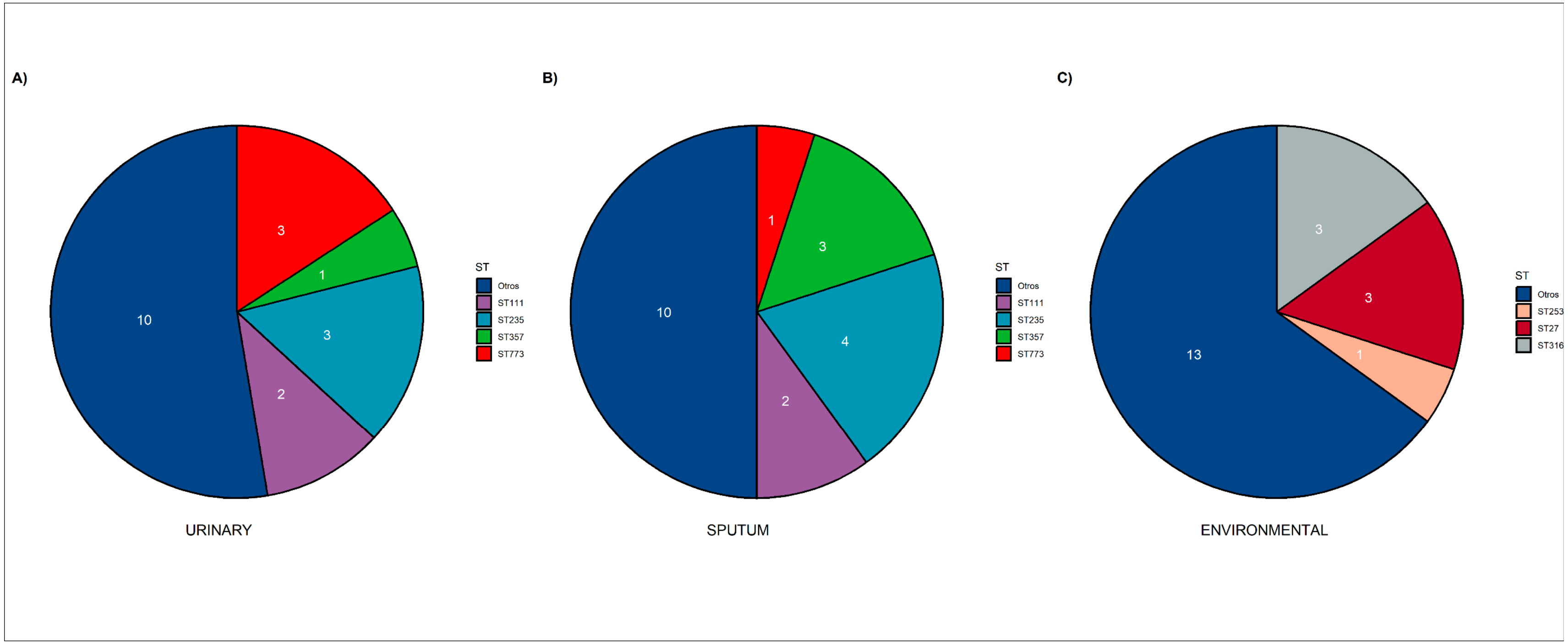

Figure S1. ST of the genomes of urinary (A), sputum (B), and environmental (C) strains. High-risk STs were detected in all three groups: being more predominant in sputum strains (75%).

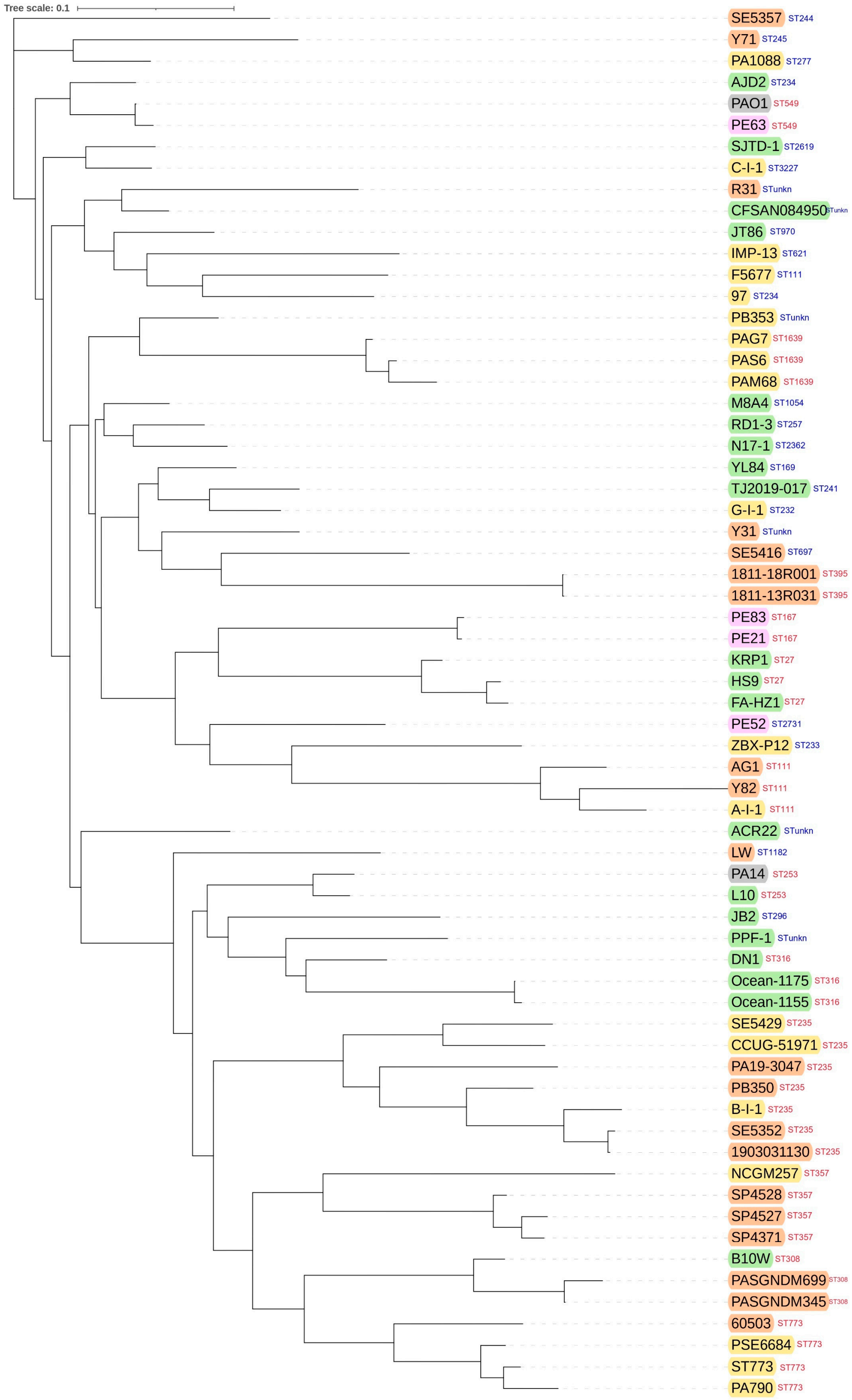

Figure S2. Phylogenetic tree based on presence and absence of accessory genome from the 65 strains. Strain names were colored according to isolation source. Yellow: urinary strains; Orange: sputum strains; Green: environmental strains; Pink: Mexican strains; Gray: Reference strains.

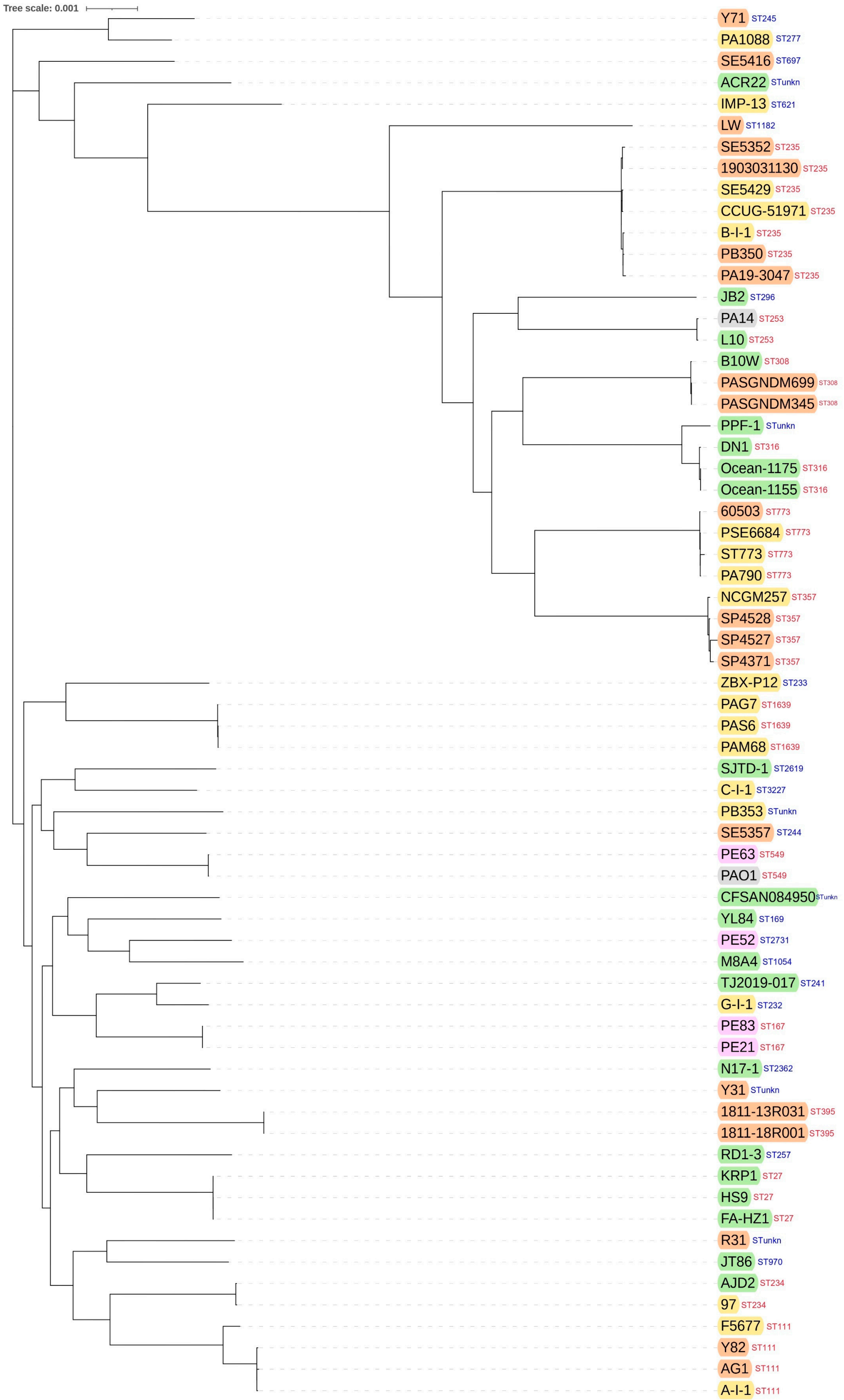

Figure S3. Dendrogram generated from the core genome. Strain names were colored according to the isolation source. Yellow: urinary strains; Orange: sputum strains; Green: environmental strains; Pink: Mexican strains; Gray: Reference strains.

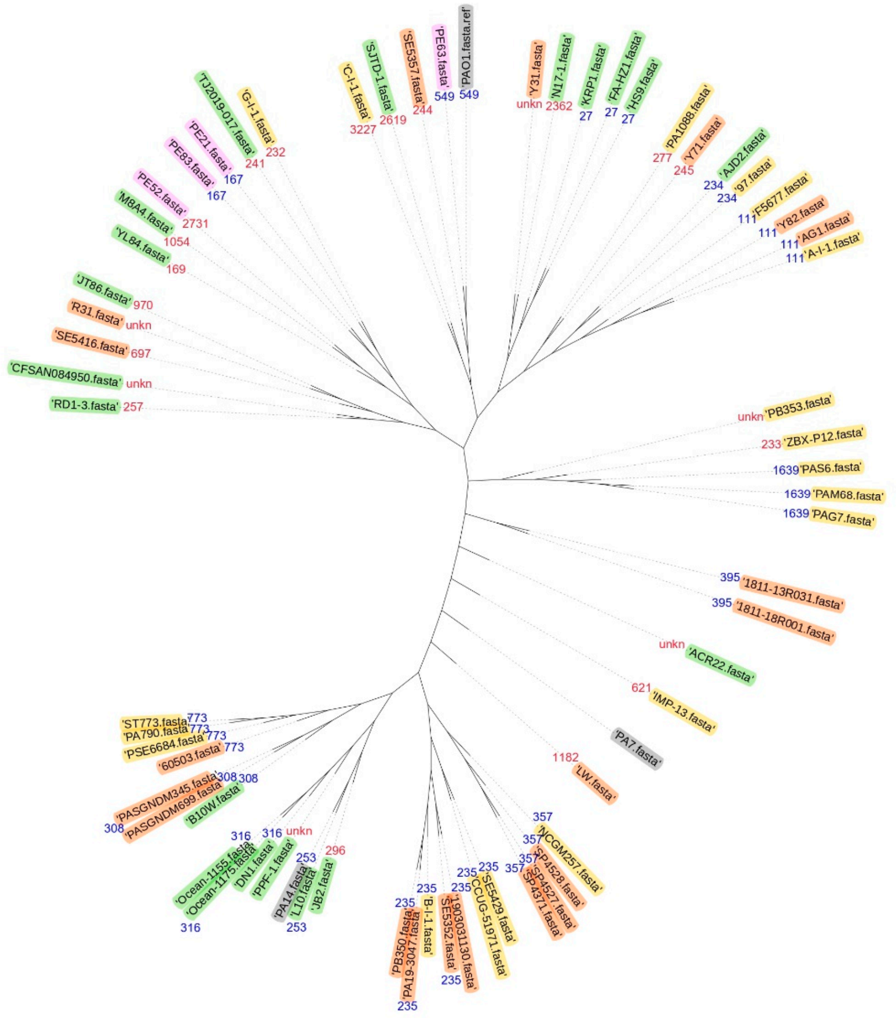

Figure S4. Phylogenetic tree generated based on SNPs. Strain names were colored according to the isolation source. Yellow: urinary strains; Orange: sputum strains; Green: environmental strains; Pink: Mexican strains; Gray: Reference strains. Numbers in colors red and blue are the STs.

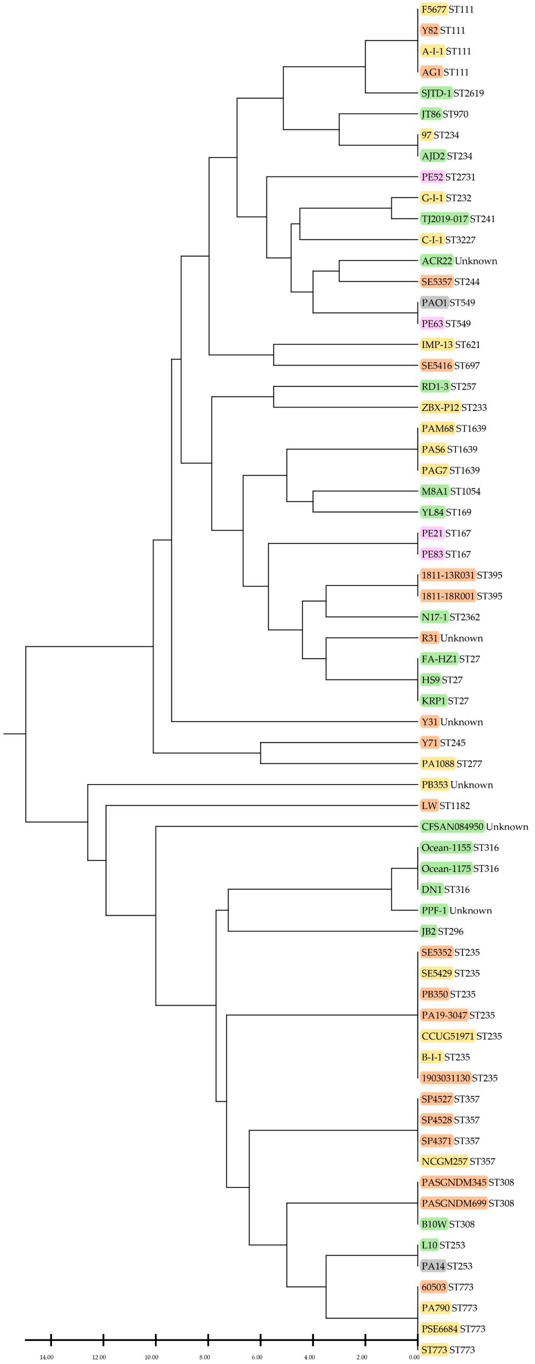

Figure S5. Phylogenetic tree based on concatenated sequences of MLST. The dendrogram was constructed with MEGA 11 using the UPGMA algorithm. Linkage distances are indicated on the scale at the bottom. Strain names were colored according to isolation source. Yellow: urinary strains; Orange: sputum strains; Green: environmental strains; Pink: Mexican strains; Gray: Reference strains.

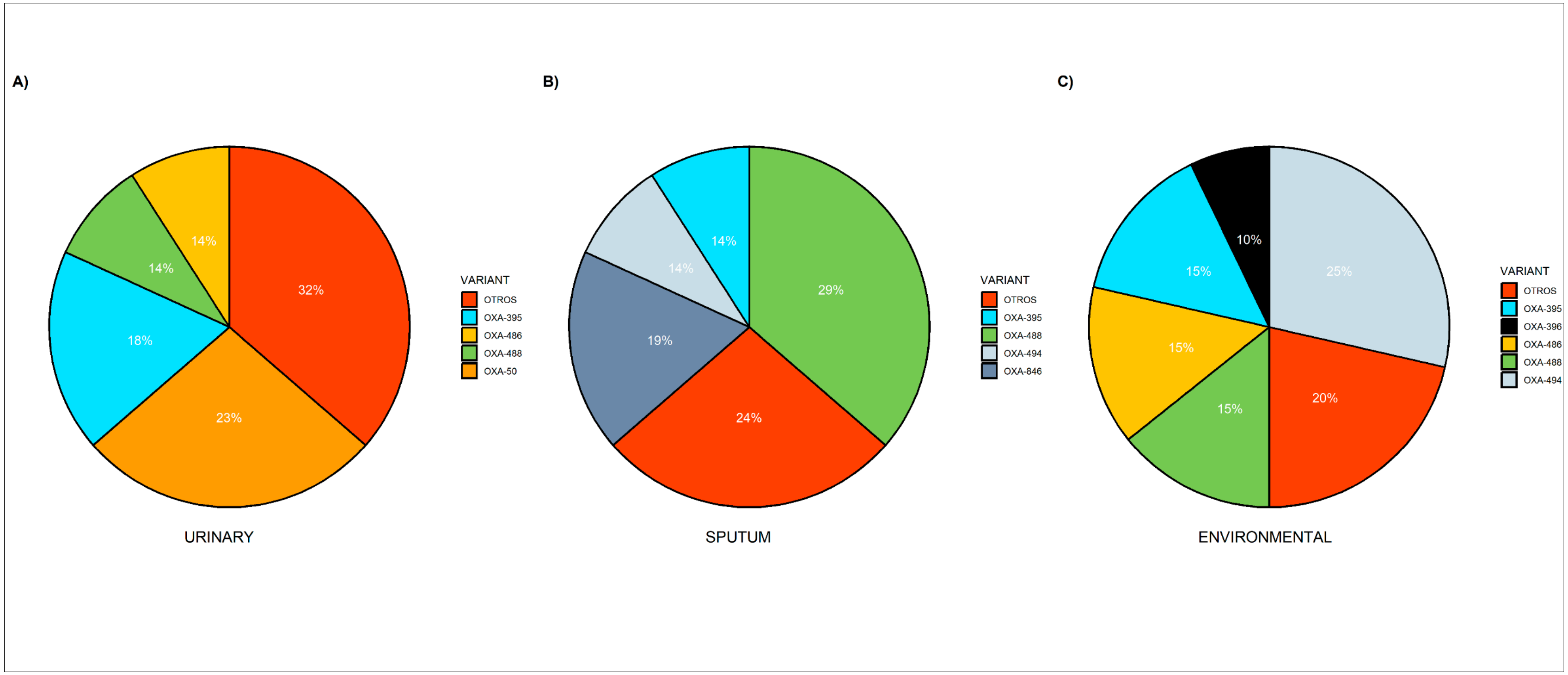

Figure S6. *bla*$_{OXA-50}$ family variants of strain genomes of A) Urinary, B) Sputum, and C) Environmental. The Beta-lactamase database was used to determine all variants of *bla*$_{OXA-50}$.

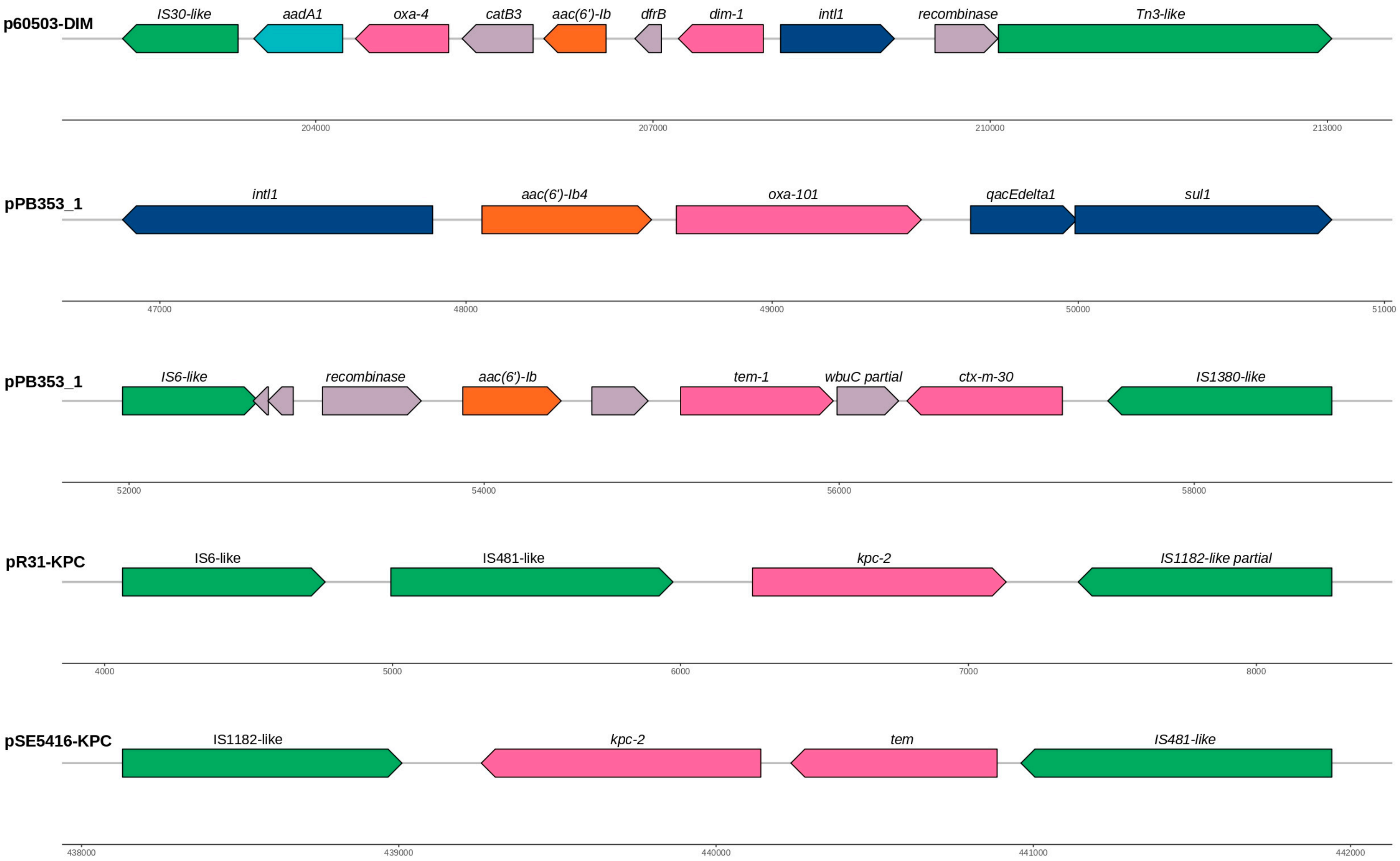

Figure S7. Mobile Genetic Elements carrying beta-lactams resistance genes found in plasmids. Blue arrows represent integron genes and green arrows represent insertion sequences. Pink arrows are the beta-lactamases and acetylases genes are represented with orange arrows. Other genes are in color gray.

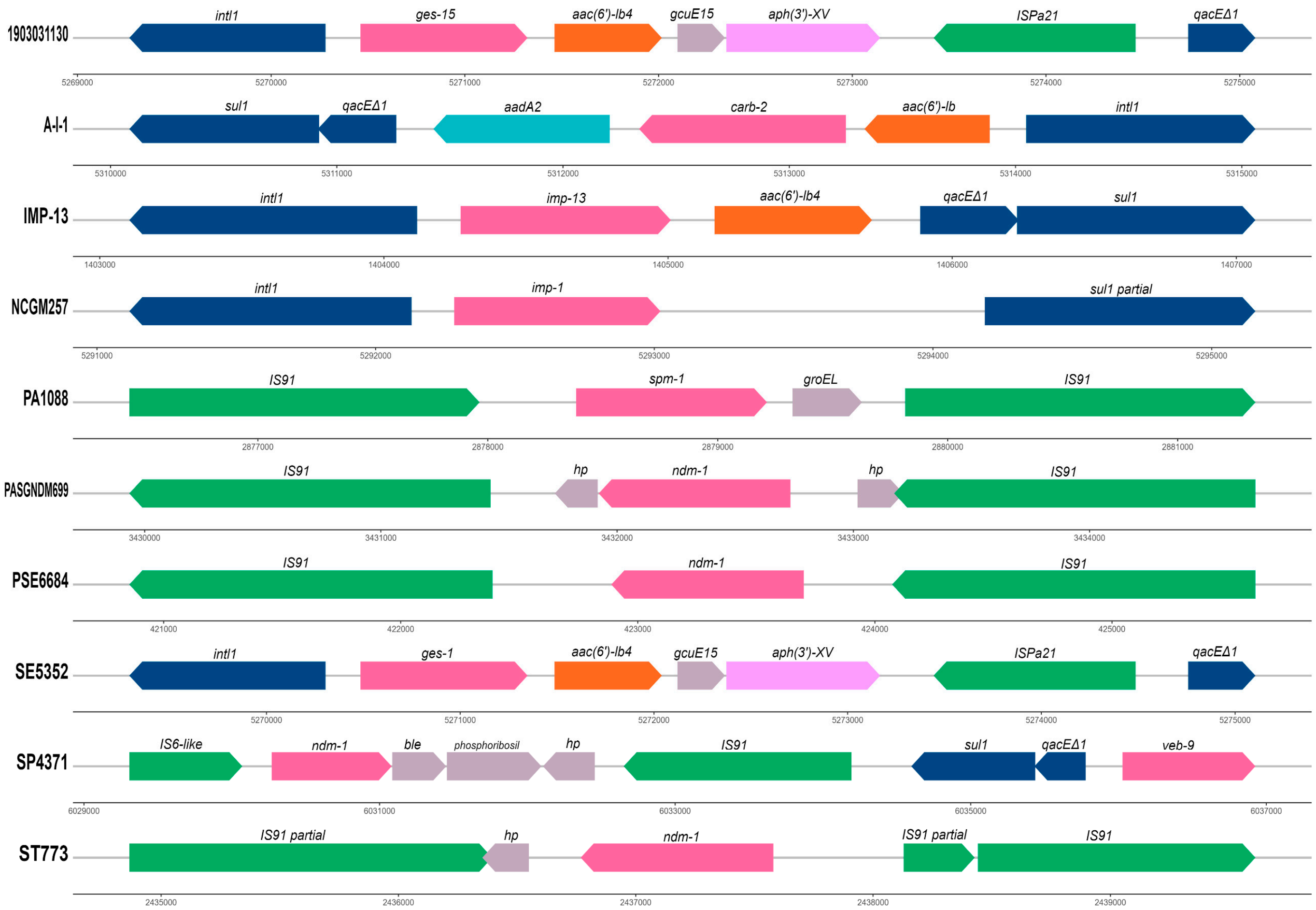

Figure S8. Mobile Genetic Elements carrying beta-lactams resistance genes found in Integrative Conjugative Elements with type 4 secretion system complete. Blue arrows represent integron genes and green arrows represent insertion sequences. Pink, yellow, orange, and dark pink arrows represent the beta-lactamases, phosphotransferases, acetylases and adenylases genes. Other genes are in color gray. This is a representative figure of some MGEs found in the genomes included in this study.

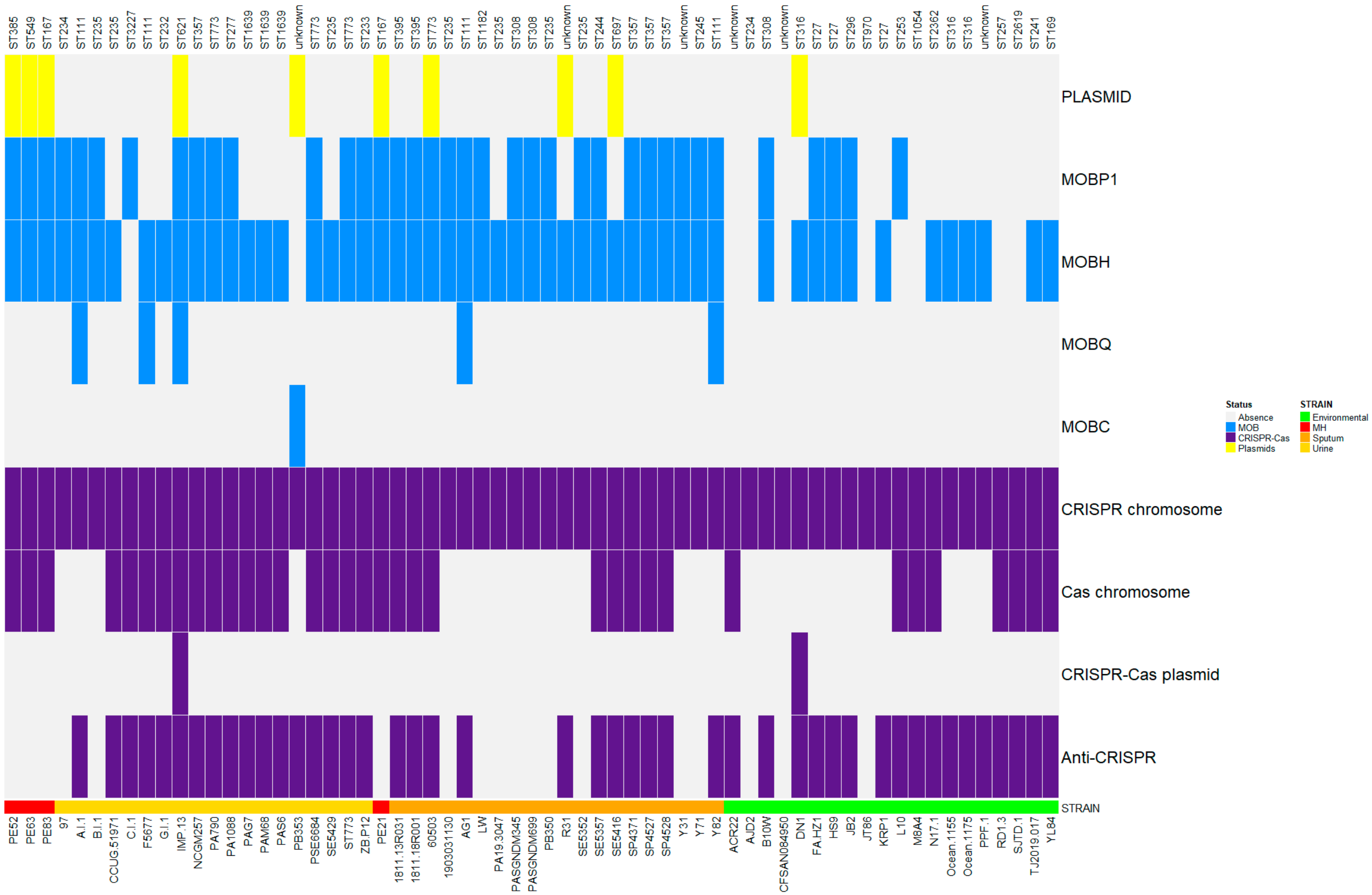

Figure S9. Presence and absence of MOB relaxases, chromosomal CRISPR-Cas systems, plasmid CRISPR-Cas systems, and anti-CRISPR systems. The heatmap represents in yellow the presence of plasmids, in blue the presence of MOB relaxases and purple the presence of the different CRISPR-Cas and ANTI-CRISPR systems. *MH means Mexican Hospital.
